# Phonemic awareness as a pathway to number transcoding

**DOI:** 10.3389/fpsyg.2014.00013

**Published:** 2014-01-28

**Authors:** Júlia B. Lopes-Silva, Ricardo Moura, Annelise Júlio-Costa, Vitor G. Haase, Guilherme Wood

**Affiliations:** ^1^Developmental Neuropsychology Laboratory, Department of Psychology, Universidade Federal de Minas GeraisBelo Horizonte, Brazil; ^2^Programa de Pós-graduação em Saúde da Criança e do Adolescente, Universidade Federal de Minas GeraisBelo Horizonte, Brazil; ^3^Programa de Pós-graduação em Neurociências, Universidade Federal de Minas GeraisBelo Horizonte, Brazil; ^4^Department of Neuropsychology, Institute of Psychology, Karl-Franzens-University of GrazGraz, Austria

**Keywords:** phonemic awareness, verbal working memory, transcoding, ADAPT, asemantic transcoding models

## Abstract

Although verbal and numerical abilities have a well-established interaction, the impact of phonological processing on numeric abilities remains elusive. The aim of this study is to investigate the role of phonemic awareness in number processing and to explore its association with other functions such as working memory and magnitude processing. One hundred seventy-two children in 2nd grade to 4th grade were evaluated in terms of their intelligence, number transcoding, phonemic awareness, verbal and visuospatial working memory and number sense (non-symbolic magnitude comparison) performance. All of the children had normal intelligence. Among these measurements of magnitude processing, working memory and phonemic awareness, only the last was retained in regression and path models predicting transcoding ability. Phonemic awareness mediated the influence of verbal working memory on number transcoding. The evidence suggests that phonemic awareness significantly affects number transcoding. Such an association is robust and should be considered in cognitive models of both dyslexia and dyscalculia.

## Introduction

Mastering reading and writing numbers in their verbal and Arabic forms is an essential skill for daily life (Lochy and Censabella, 2005). Being able to manipulate numbers and convert them from one format into another is one of the first steps in children's mathematical learning and starts to be formally trained in kindergarten. The ability to establish a relationship between the verbal and Arabic representations of number, when a conversion of numerical symbols from one notation to the other is necessary, is called number transcoding (Deloche and Seron, 1987).

The verbal number system is linguistically structured and, although it may differ among languages, there are some common basic principles and regularities (Fayol and Seron, 2005). It is typically composed of a lexicon of single words that designate a few quantities (like *five, eleven, seventy* and *hundred*) and organized by a syntax that arranges these lexical units in order to represent any possible quantity. The two basic syntactic principles are the relations of addition and multiplication. In this sense, numbers are represented as sum relationships (e.g.: *eighty-one* means *eighty* plus *one*) and product relationships (e.g.: *three hundred* means three times *hundred*). The number words in Portuguese are similar to the English number words in the sense that they are also organized in lexical classes for units, decades and particulars (the *-teens* in English) (Wood et al., 2006).

The Arabic code is more complex and is acquired later in development (Geary, 2000). Its lexicon is composed of only a small set of different symbols (digits from 0 to 9), and the basic syntactic principle that combines them to form all numbers is the positional value (or place-value). According to this principle, the digit's value depends on its position in the numerical string and is given by a power of base ten. Therefore, in the case of three-digit numbers, the first digit (from right to left) is multiplied by 10^0^, the second by 10^1^, and so on. The number 124, for example, represents a quantity equal to 1 × 10^2^ + 2 × 10^1^ + 4 × 10^0^ (or 100 + 20 + 4). The digit 0 has a special syntactic role when it denotes the absence of a given power of ten, as occurs in numbers with internal zeros, for example the number 406 (4 × 10^2^ + 0 × 10^1^ + 6 × 10^0^).

One preeminent model of number transcoding is ADAPT (A Developmental, Asemantic, and Procedural model for Transcoding from verbal to Arabic numerals; Barrouillet et al., 2004). According to ADAPT, the inputs are coded into a phonological sequence and the parsing mechanisms then subdivide this sequence into smaller units to be processed by a production system. This production system is related to rules devoted to the retrieval of Arabic forms from long-term memory (LTM) (called P1 rules), to managing the size of digit chains (P2 and P3 rules, which create a frame of two or three slots) and to filling these slots (if there are any empty slots, P4 rules will fill them with 0s). Separators, such as thousands and hundreds, are used to identify the number of slots; once every segment is placed in its digit form in the chain, it is transcribed. The model accounts for the development of transcoding processes through practice: experience leads to an expansion of the numerical lexicon and improvement of conversion rules.

The ADAPT model is the only cognitive model of number transcoding which makes testable predictions regarding both working memory capacity and phonological/lexical representations and their respective roles in the typical and atypical development of transcoding abilities. Moreover, even though it is not explicitly stated in the original publication (Barrouillet et al., 2004), ADAPT clearly emphasizes the importance of phonological encoding in the first steps of number writing production, and this has not been investigated in more detail. Because both working memory and the ability to form lexical representations of numbers and - as we assume here - phonemic awareness are related to mathematical performance, ADAPT is the only transcoding model directly examined in the present study.

Short-term memory and working memory (thereafter WM) are involved in the temporary storage of verbal information, lexical retrieval, and the execution of the manipulations to generate the Arabic output. Working memory representations are also involved in creating a sequence of digits and possibly blank spaces to be filled with subsequent procedures. It has been consistently related to number transcoding performance and error patterns (Camos, 2008; Zuber et al., 2009; Pixner et al., 2011). The role of working memory in transcoding tasks can be outlined in the following steps: encoding the number to be transcoded; monitoring the application of transcoding rules and the production of the numeral (Lochy and Censabella, 2005).

Another cognitive mechanism that may be involved in number transcoding is phonemic awareness. Phonemic awareness is the subcomponent of phonological processing which is related to the ability to perceive and manipulate the phonemes that constitute words (Wagner and Torgesen, 1987). According to the ADAPT model (Barrouillet et al., 2004), the phonological encoding of the verbal numerals is the primary step in transcoding procedures, before the use of algorithm rules and retrieval from LTM. Therefore, limitations in phonological processing capacity may constrain the ability to transcode, particularly in the case of longer and more complex numbers. Phonological processing may also interact with the capacity of verbal working memory. The more demanding the phonological processing of numerical stimuli, the fewer resources would remain available in verbal working memory for transcoding. Although the conversion of a verbal representation to an Arabic one is related to phonological representations, this association has not yet been investigated in detail in the ADAPT model.

Krajewski and Schneider (2009) found that phonological awareness facilitates the differentiation and manipulation of single words in the number word sequence. These authors built a model of early arithmetic development that postulates three different levels: (1) basic numerical skills, in which children are already able to discriminate between quantities and to recite number words, without accessing their quantitative semantic meaning; (2) quantity-number concept, when there is a linkage between magnitudes and the number words that represent them; (3) number relationships, the point at which children understand that the difference between two numbers is another number. According to these authors, phonological awareness (measured by phoneme synthesis and rhyming tasks) plays an important role in the first level. The authors claim that because this phonological skill is related to the ability to differentiate and manipulate meaningful segments of language, it is also important in differentiating number words (“one,” “two,” “three” instead of “onetwothree”).

In view of the above, the aim of this study is to investigate the role of specific cognitive mechanisms underlying number transcoding such as general cognitive ability, verbal and non-verbal short-term and working memory, magnitude representation, and phonemic awareness. More specifically, our main goal was to investigate the relative impact of phonemic awareness on number transcoding. Phonemic awareness is related to reading and spelling skills (Wagner and Torgesen, 1987; Castles and Coltheart, 2004; Hulme et al., 2012; Melby-Lervå et al., 2012), and recent studies have also focused on its association with arithmetic fact retrieval and with arithmetic word problems (Hecht et al., 2001; Boets and De Smedt, 2010; De Smedt et al., 2010). Importantly, many measures of phonemic awareness, such as the phoneme elision task employed in the present investigation, require a certain availability of working memory resources. Working memory is recruited in such tasks when the participant must hold a word in mind while determining the phonological information to be deleted (De Smedt et al., 2010). Both verbal and visuospatial working memory play important roles in numerical transcoding according to the ADAPT model (Camos, 2008; Zuber et al., 2009), but no study so far has investigated the specific contribution of phonemic awareness and working memory in number transcoding tasks.

Two main hypotheses will be addressed in the present study: First, based on the central role assigned by the ADAPT model to working memory capacity (Barrouillet et al., 2004; Camos, 2008), one can argue that working memory contributes to number transcoding independently because working memory capacity is putatively implicated in the use of transformation rules and procedures employed during transcoding. Second, at least part of the influence of working memory on number transcoding should be mediated by phonemic awareness. Phonemic awareness scores are assumed to index the quality of the underlying phonological representations. These representations are related to the perception and manipulation of sound-based processes (Simmons and Singleton, 2008); therefore, phonemic awareness performance would have an impact on verbal working memory and transcoding skills.

## Materials and methods

The study was approved by the local research ethics committee (COEP–UFMG) and is in line with the Declaration of Helsinki. Children participated only after informed consent was obtained. Informed consent was obtained in written form from parents and orally from children.

### Sample

A total of 487 children in grades 2–4 were invited from public schools in Belo Horizonte, Brazil. Of these children, 207 (42%) children agreed to take part in this study. Testing was conducted in the children's own schools. The various tasks were presented in four different pseudo-random orders during one session that lasted approximately 1 h.

We excluded five children from the sample due to low intelligence (performance on Raven's Colored Progressive Matrices below one standard deviation). One child did not complete the entire battery and was also excluded from the analysis. Twenty-nine children were excluded from further analyses because either they had a poor R^2^ on the fitting procedure to calculate their internal Weber fraction on the non-symbolic comparison task (*R*^2^ < 0.2) or they showed an internal Weber fraction that exceeded the limit of discriminability of the non-symbolic magnitude comparison task (*w* > 0.6). The final sample comprised 172 children (55.2% girls), with a mean age of 111.84 months (*SD* = 10.90), ranging from 94 to 140 months.

### Instruments

The following instruments were used in the cognitive assessment: Raven's Colored Progressive Matrices, Digit Span, Corsi Blocks, Non-symbolic magnitude comparison task, Phoneme Elision and Arabic number writing task.

- Raven's Colored Progressive Matrices: general intelligence was assessed with the age-appropriate Brazilian validated version of Raven's Colored Matrices (Angelini et al., 1999). The analyses were based on z-scores calculated from the manual's norms.
- Digit Span: Verbal short-term and working memory were assessed with the Brazilian WISC-III Digit Span subtest (Figueiredo, 2002). Performance in the forward order was considered a measure of verbal short-term memory, and the backward order was used to assess verbal working memory (Figueiredo and Nascimento, 2007). We evaluated the total score (correct trials x span) in both the forward and backward orders.
- Corsi Blocks: This test is a measure of the visuospatial component of short-term and working memory. It consists of a set of nine blocks, which the examiner taps in a certain sequence. The test starts with sequences of two blocks and can reach a maximum of nine blocks. We used the forward and backward orders according to Kessels et al. (2000). In the forward condition, the child is instructed to tap the blocks in the same order as the examiner, and in the backward condition, in the reverse order. We also evaluated the total scores.
- Non-symbolic magnitude comparison task: In this task, the participants were instructed to compare two simultaneously presented sets of dots, indicating which one contained the larger number. Black dots were presented on a white circle over a black background. In each trial, one of the two white circles contained 32 dots (reference numerosity) and the other contained 20, 23, 26, 29, 35, 38, 41, or 44 dots. Each magnitude of dot sets was presented eight times. The task comprised 8 learning trials and 64 experimental trials. Perceptual variables were varied such that in half of the trials the individual dot size was held constant, while in the other half, the size of the area occupied by the dots was held constant (see exact procedure descriptions in Dehaene et al., 2005). Maximum stimulus presentation time was 4.000 ms, and the inter-trial interval was 700 ms. Before each trial, a fixation point appeared on the screen: a cross, printed in white, with each line 30 mm long. If the child judged that the right circle presented more dots, a predefined key localized in the right side of the keyboard should be pressed with the right hand. However, if the child judged that the left circle contained more dots, then a predefined key on the left side had to be pressed with the left hand (Costa et al., 2011). As a measure of the number sense acuity, the internal Weber fraction (w) was calculated for each child based on the Log-Gaussian model of number representation (Dehaene, 2007), with the methods described by Piazza et al. (2004).
- Phoneme Elision: This is a widely accepted measure of phonemic awareness (Wagner and Torgesen, 1987; Castles and Coltheart, 2004; Hulme et al., 2012; Melby-Lervå et al., 2012). The child hears a word and must say what the word would be if a specified phoneme in the word were to be deleted (e.g., “*filha*” without /f/ is “*ilha*” [in English, it would be similar to “cup” without /k/ is “up”). The test comprises 28 items: in 8 items, the child must delete a vowel, and in the other 20, a consonant. The consonants to be suppressed varied by place and manner of articulation. The phoneme to be suppressed could be in different positions within the words, which ranged from 2 to 3 syllables. The internal consistency of the task is 0.92 (KR-20 formula).
- Arabic number writing task: To evaluate number transcoding, children were instructed to write the Arabic forms of dictated numbers. This task consists of 40 items, up to 4 digits (3 one-digit numbers, 9 two-digit numbers, 10 three-digit numbers and 18 four-digit numbers). The one- and two- digit numbers were classified as “lexical items” (12 items), and the other 28 items require the use of algorithm-based rules in order to be written (Barrouillet et al., 2004; Camos, 2008). This task has been used in a previous study with a comparable sample, and the consistency of this task was KR-20 = 0.96 (Moura et al., 2013).

### Analysis

The differential impact of phonemic awareness and working memory on number transcoding was investigated in a hierarchical regression analysis with Arabic number writing as the dependent variable. Age and intelligence were entered first, and working memory and the Weber fraction in a second step, using the stepwise method. The phoneme elision task was entered in the model in a third step, also using the stepwise method. This allowed us to investigate the specific contribution of phonemic awareness to number transcoding performance after working memory variance was taken into account.

As a complement, path analyses, including all measures of age, intelligence, working memory and phonemic awareness were calculated, to determine the specific contribution of phonemic awareness as a mediator of the effect of working memory on number transcoding.

## Results

Thirty-three percent of the children did not commit any errors in the number transcoding task. Ninety-three percent of the children did not commit any errors on the numbers that can be lexically retrieved (items 1–12). According to what is suggested by the ADAPT model, errors rates increased with the number of rules required for number transcoding. In the numbers that required 3 transcoding rules, 50% of the children committed errors, in the 4-rules, 71.6% presented some errors, in the 5-rules, 73.3% and, finally in the more complex items (6 and 7 rules), 84.5% of the children committed, at least, one error.

Since one-third of the sample did not commit any error in the transcoding task, one may argue that they should be excluded from the sample to avoid biases in the estimation of the covariance matrix, particularly with regard to the association between transcoding performance and other cognitive functions. To investigate the occurrence of bias, regression and path analyses were performed in the full sample and in the sample without the children with perfect score in the transcoding task. Results were numerically comparable in both regression and path analyses and their interpretation was exactly the same. For this reason, we decided to report the results obtained by analysing the full sample.

### Association between cognitive variables and transcoding ability

First, the specific impact of the different cognitive mechanisms on number transcoding was evaluated by means of hierarchical regression models. To approximate a normal distribution, error rates of the Arabic number writing task were arcsine transformed. Initially, we examined the general association between these measures through Pearson's correlations. Inspection of Table 1 reveals that the error rates observed in the number transcoding task were negatively correlated to age, intelligence, working memory, and phonemic awareness. There was also a weak positive correlation between error rates in number transcoding and the Weber fraction, which may reflect the maturation level of more general numerical skills. Moreover, phonemic awareness was significantly correlated to intelligence and working memory.

**Table 1:** **Correlation coefficients**.

	**1**	**2**	**3**	**4**	**5**	**6**	**7**	**8**
1. Age (in months)	1							
2. Raven	−0.23^**^	1						
3. Digit span-Forward	0.19^**^	0.19^**^	1					
4. Digit span-Backward	0.05	0.34^**^	0.18^**^	1				
5. Corsi blocks Forward	0.19^**^	0.28^**^	0.15^**^	0.20^**^	1			
6. Corsi blocks Backward	0.01	0.34^**^	0.14	0.36^**^	0.36^**^	1		
7. Weber fraction	−0.19^**^	−0.11	−0.17^**^	−0.19^**^	−0.16^**^	−0.13	1	
8. Phoneme elision	0.11	0.36^**^	0.23^**^	0.36^**^	0.24^**^	0.25^**^	−0.13	1
9. Number transcoding	−0.11	−0.17^**^	−0.11	−0.15^**^	−0.10	−0.13	0.21^**^	−0.36^**^

** *Correlation is significant at the 0.01 level (2-tailed)*.

* *Correlation is significant at the 0.05 level (2-tailed)*.

To investigate in more detail the specific impact of phonemic awareness on transcoding abilities, a hierarchical regression model was calculated (Table 2). In this model, more general determinants of cognitive development were entered first, and more specific predictors of transcoding ability were included later on, in a hierarchical fashion. In step 1, age and intelligence were included as general factors that predict school achievement, using the enter method. In step 2, the following cognitive measures were included: Weber fraction and the total scores of the forward and backward orders of Digit Span and Corsi Blocks. Last, in step 3, we included the phoneme elision score. The stepwise method was used in steps 2 and 3 to avoid redundancy and to guarantee a high degree of parsimony.

**Table 2:** **Regression analysis for number transcoding (errors arcsine, adjusted *r*^2^ = 0.41)**.

**Predictor**	**Beta**	**Partial t**	**Sig**	***r*^2^ change**
Intercept		10.14	<0.001	
Age (months)	−0.404	−6.487	<0.001	0.305
Raven	−0.225	−3.282	0.001	
Digit span−backward	−0.089	−1.358	0.176	Excluded
Weber fraction	0.095	1.545	0.124	Excluded
Digit span−forward	−0.056	−0.885	0.378	Excluded
Corsi blocks−backward	−0.035	−0.529	0.598	Excluded
Corsi blocks−forward	−0.003	−0.051	0.959	Excluded
Phoneme elision	−0.337	−5.038	<0.001	0.088

The regression model reveals that after removing the effects of age and intelligence in step 1, verbal working memory remains a significant predictor of transcoding performance in step 2. Nevertheless, the addition of phonemic awareness to the model in step 3 leads to the exclusion of verbal working memory. Phonemic awareness, along with age and intelligence, was a significant predictor of number transcoding and absorbed the impact of verbal working memory on transcoding performance. The model explains a moderate amount of variance (Table 2). Measures of the approximate number system, visuospatial short-term memory, and visuospatial working memory were not retained in the model.

The reason to employ a hierarchical regression model in this analysis is to demonstrate the validity of the present experimental setup. By entering the measures of working memory in the regression model first we are able to replicate previous studies and thereby show that our measures of working memory were well-chosen and are associated to transcoding abilities. After completing this step of validation of well-established results, we continue the investigation showing that phonemic awareness absorbs the impact of measures of working memory on transcoding capacity. We have also calculated a regression model allowing the effect of phonemic awareness to vary simultaneously to measures of working memory, that is, with no hierarchical distinction between these variables. Results were largely comparable with those reported previously: only phonemic awareness is retained in the model along with intelligence and age (*R*^2^ = 0.64; adjusted *R*^2^ = 0.40; *b* = −0.02).

### Describing the roles of phonemic awareness and verbal memory in arabic number transcoding

As shown in the previous section, the influence of the verbal working memory on number transcoding is shared with phonemic awareness. Therefore, as a complement to the previous findings, path analyses including both working memory and phonemic awareness, as well as Weber fraction, were calculated in order to investigate the interplay of these variables in number transcoding.

To determine the strength of the effect of phonemic awareness on number transcoding, a sequence of models was calculated and compared. Chi-square and the approximate fit indexes root mean square residual (RMR), goodness of fit index (GFI), adjusted goodness of fit index (AGFI), comparative fit index (CFI) and root mean square error of approximation (RMSEA) were used to evaluate model quality. A non-significant chi-square indicates no significant discrepancy between model and data. The RMR measures the ratio of residuals in comparison to the covariances expressed by the models. Values smaller than 0.10 are considered adequate. GFI, AGFI, and CFI evaluate the degree of misspecification present in the model. Usually, the best acceptable values are greater than 0.90. Finally, the Root Mean Square Error of Approximation, or RMSEA, considers the model complexity when evaluating the model fit. The RMSEA is considered acceptable when it is lower than 0.05. The Chi-square difference between models was employed to compare models with increasing numbers of free parameters. Models were calculated in the software AMOS v.19 using the maximum likelihood estimation function.

**Figure 1:**
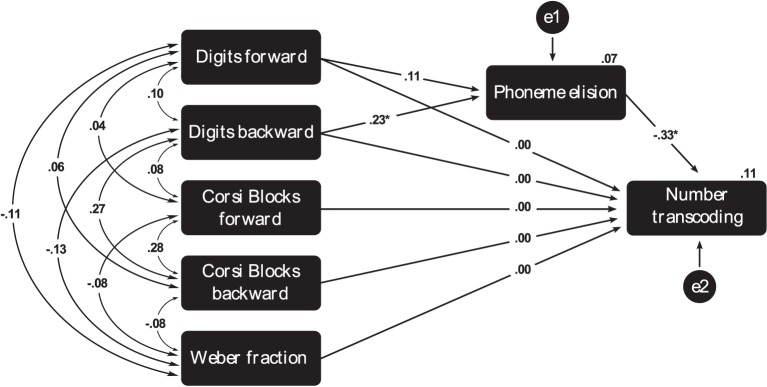
**Path-analysis model describing the effects of working memory, Weber fraction and phonemic awareness in a number transcoding task**. Paths marked with ^*^ are significant at the level 0.05 and with ^**^ are significant at the level 0.001.

To control for the influence of developmental and intellectual levels on the path models, we calculated the unstandardized residuals of the independent variables (short-term and working memory, Weber fraction and phonemic awareness), in which the portion of variance due to age (in months) and/or intelligence was removed. These adjusted values of working memory, magnitude processing and phonemic awareness were entered as the exogenous variables in the path analyses. All the covariances between the exogenous variables were set as free (Figure 1).

Those variables with negative standardized values indicate that higher scores in these predictors lead to lower error rates in the number transcoding task. The only exception is the Weber Fraction path, in which higher values indicate poorer magnitude representation acuity and, hence, more errors in number transcoding.

Fit statistics of path models are shown in Table 3. The first and most complex model (ALL PATHS) included the two measures of short-term and working memory (forward and backward versions of Digit Span and Corsi Blocks), as well as Weber fraction and an additional Phoneme Elision mediation path between both the forward and backward versions of the Digit Span and the number writing tasks. This model presented adequate fit indexes but is not parsimonious. Models with fewer parameters to be estimated were designed and were compared to the ALL PATHS model and to one another.

**Table 3:** **Fit statistics for path models regarding number writing performance**.

**Number transcoding model**	**χ2**	***df***	***p***	**RMR**	**GFI**	**AGFI**	**CFI**	**RMSEA**
ALL PATHS	0.793	3	0.851	1.066	0.999	0.988	1.000	0.000
NO VISUOSPATIAL	0.953	5	0.966	1.067	0.998	0.991	1.000	0.000
NO ANS	2.396	6	0.880	1.067	0.996	0.981	1.000	0.000
MEDIATION PATH	4.156	8	0.843	1.071	0.993	0.976	1.000	0.000
NO MEDIATION	30.654	9	<0.001	3.388	0.954	0.858	0.563	0.119

*Note: RMR, root mean square residual; GFI, goodness-of-fit index; AGFI, adjusted-goodness-of-fit index; CFI, comparative fit index; RMSEA, root mean square error of approximation*.

First, the NO VISUOSPATIAL model removed the paths from visuospatial memory to transcoding. Accordingly, the NO ANS model also suppressed the path from the Weber fraction to transcoding. In one further step, two models were calculated. In the first (MEDIATION PATH), the contribution of verbal working memory to transcoding is partially mediated by phonemic awareness. Finally, to determine the relevance of phonemic awareness for transcoding, in the last model, the path from Phoneme elision to Number transcoding was removed, while the direct paths from verbal working memory to transcoding were retained (NO MEDIATION). If the exclusion of any of these paths leads to a statistically significant decrease in model fit, one may conclude that the specific parameters removed from the more parsimonious version of the path model contribute substantially to model fit.

Inspection of Table 3 reveals that all models including the Phoneme Elision-mediation path reached satisfactory fit levels. Nevertheless, all models presented large residuals, as indicated by the RMR, which suggests that the variables included in the models were not sufficient to fully explain the variance in the number writing task. However, non-significant Chi-squares and the other fit measures associated with these models were largely acceptable.

Overall, the model that presented the worst fit indices was the one that excluded the Phoneme Elision-mediation path and assumed that Digit Span has a direct influence on number transcoding (NO MEDIATION). Model comparisons corroborate these results because the model NO MEDIATION presented statistically poorer fit than all other models. Its chi-square was statistically significant, and the model did not present any adequate fix indexes (Table 3). This finding suggests that phonemic awareness is a relevant predictor of transcoding performance, with substantial specific contribution. Moreover, comparisons among all other models only produced non-significant chi-square differences. Given the statistical equivalence of these models, one may select the model MEDIATION PATH, in which the effect of working memory on transcoding performance is partially mediated by phonemic awareness, as the most parsimonious description of the present data. Importantly, the association between verbal working memory and phonemic awareness is stronger than that between verbal short-term memory and phonemic awareness. Regression values of the model MEDIATION PATH are depicted in Figure 1.

## Discussion

The present study investigated the impact of phonological skills on a number transcoding task, and it is, to our knowledge, the first to simultaneously evaluate the relative impact of short-term and working memory, number sense and phonemic awareness on number transcoding. Our results revealed two main findings. First, we confirmed previous evidence of a verbal working memory effect on number transcoding, and, more importantly, we provided evidence of a relationship between number transcoding and phonemic awareness. Our second main finding is that the well-established relationship between verbal working memory capacity and number transcoding is mediated by phonemic awareness abilities. In the following sections, these topics will be discussed in more detail.

### The impact of verbal and visuospatial working memory on arabic number writing

The performance of children in the number writing task was far from being flawless. They present many errors on the more complex two-, three-, and four-digit items, which require more than three transcoding rules, according to ADAPT. These findings are in accordance to what has been reported in the literature regarding transcoding skills of school aged children (Moura et al., 2013) and have been interpreted as a product of working memory processes in number transcoding (Camos, 2008). However, little is effectively known about the selective impact of different components of working memory on number transcoding. To our knowledge, this was the first study to analyze this problem in greater depth. Although a specific role of the central executive function in transcoding has been suggested (Camos, 2008), the present study is the first to explore the impact of phonological and visuospatial working memory in a number writing task and distinguish them from the central executive. We provide evidence regarding the specific role of phonological working memory and, more precisely, of the quality of underlying phonological representations, by means of the phonemic awareness performance.

Working memory plays an important role in the algorithmic-based procedures of number transcoding (Camos, 2008; Pixner et al., 2011). Essentially, it is believed to be involved in the maintenance of verbal units from the verbal numbers and in managing the new digit chain. In our study, we found that better verbal working memory capacity was associated with higher number transcoding performance. Interestingly, the same does not apply to the visuospatial components of short-term and working memory, as none of them revealed an association with transcoding performance in correlation, regression or path analyses. In a previous study by Zuber et al. (2009), the visuospatial working memory component was associated with the management of Arabic code syntax. Nevertheless, it is important to note here that the sample used in this other study was composed of German-speaking first graders, and the German number word system is different from the Portuguese system. In German, the order of the units and decades in the verbal numerals is inverted in comparison to the Arabic ones. One possibility, therefore, is that transcoding numbers in Portuguese demands less visuospatial working memory capacity than in languages with this inversion. Linguistic comparison research remains necessary to confirm this hypothesis.

Raghubar et al. (2010) reviewed evidence indicating that the influence of the subcomponents of working memory on arithmetic performance might vary according to age. The visuospatial component is recruited in earlier phases of development, while children are still learning basic mathematical concepts, whereas the phonological loop is more relevant after these skills have already been mastered. Although Raghubar et al. (2010) did not specifically discuss number transcoding, this study reviews evidence regarding the complex and dynamic nature of the relationship between working memory and math achievement. Consistent with these results, no effect of visuospatial working memory on number transcoding was observed in second- to fourth-grade children in the present study.

### The relationship between verbal working memory and phonemic awareness

The first step of writing Arabic numbers from dictation proposed by the ADAPT model (Barrouillet et al., 2004) is the phonological encoding of the auditory input, which consists of verbal numerals. Nevertheless, the procedures involved in this phonological encoding are still not completely specified. Here we showed that, in addition to working memory capacity, phonemic awareness also plays an important role in number transcoding. Our results showed that even when considering the influence of working memory and basic numerical skills on number transcoding, the predictive value of phonemic awareness abilities was substantial. This suggests that phonemic awareness is an important facilitator of the phonological encoding required in the initial steps of number transcoding.

Another aim of the present study was to clarify the influence of phonemic awareness on number transcoding. We aimed to investigate whether there is a direct influence of verbal working memory on number transcoding or if this association would be mediated by phonemic awareness. Our results presented evidence showing that phonemic awareness mediates the influence of verbal working memory in number transcoding, even after controlling for the effects of age and intelligence. In the path analyses, the removal of the Phoneme Elision-mediation path had a deleterious effect on model fit, which suggests that this parameter contributes crucially to improve the model fit.

This finding is consistent with the ADAPT model, which postulates that the first step in number transcoding would be the encoding of the verbal string into its phonological form (Barrouillet et al., 2004). This encoding phase would be followed by parsing procedures that segment these strings into smaller units. Smaller units are then sequentially processed through a production system in which verbal working memory is required for transcoding algorithms. It is possible to hypothesize that phonemic awareness would be the main cognitive precursor engaged in the phonological encoding phase that precedes further verbal working memory involvement in number transcoding.

A plausible explanation for the association between phonemic awareness and the influence of verbal working memory in number transcoding is the “weak phonological representation hypothesis” (Simmons and Singleton, 2008). According to this model, phonological processing deficits would impair the quality of phonological representations and thus affect aspects of numerical cognition that involve the manipulation of a verbal code.

The performance in verbal working memory and phonemic awareness depend on the same underlying and latent phonological representations (Hecht et al., 2001; Alloway et al., 2005; Durand et al., 2005). In our study, it was also possible to observe this association through the positive correlation between verbal working memory and phonemic awareness. Baddeley et al. (1975) had already suggested that, given that verbal short-term memory is a speech-based system, its capacity should be measured in more basic speech units, such as phonemes. Oakhill and Kyle (2000) also found that phonemic awareness (operationalized by means of phoneme elision and phoneme segmentation tasks) had a strong association with word and sentence span.

Evidence indicates that the influences of phonemic awareness and verbal working memory on literacy acquisition are both shared and unique (Mann and Liberman, 1984; Alloway et al., 2005). Factor analytical studies indicate that different types of phonological awareness tasks are loaded onto a single latent construct (Schatschneider et al., 1999). Tasks vary, however, in the additional cognitive demands they impose, regarding, for instance, working memory and other general cognitive components. According to this type of reasoning, different phonemic awareness tasks assess a common phonological processing construct plus additional varying components that change according to task demands. A task such as phoneme elision would consist then of at least two components, one tapping the phonological latent construct and the other one depending on working memory demands. Previous studies (Oakhill and Kyle, 2000; Alloway et al., 2005) have investigated the influence of verbal working memory on phonemic awareness performance. This question, however, is rather complex and our results emphasize the importance of also investigating the other direction of this relationship. This is especially relevant regarding the interplay between verbal working memory, phonemic awareness and number transcoding skills.

Another dimension adding complexity to the relationship between phonemic awareness and verbal working memory is the child's individual level of development, which may be characterized as the degree of automatization in phonological processing. Before the child acquires expertise with phonemic awareness, a task such as phoneme elision may impose heavy demands on the central executive. As the child progressively acquires experience with phonological processing, this task can be solved in a more automatic way, freeing working memory resources for other tasks relevant for more advanced operations. If, however, the child does not acquire abilities of accurately and automatically processing the phonemic units, precious working memory resources will be less available for numerical transcoding. Accurate and automatic phonemic processing liberates sparse processing resources necessary to solve more complex tasks.

Disclosing a complex relationship among working memory, phonemic awareness and transcoding has important consequences for math achievement in general and for its disorders. School achievement in reading and/or mathematics depends on a complex interaction between general and specific cognitive factors. As the child acquires expertise in specific domains, such as phonemic and/or quantitative representations, processing resources are liberated to work in increasingly more complex activities. The accurate and automatic nature of more basic sound and quantitative representations may thus influence the whole process of school learning, explaining variances both in achievement and in working memory. Johnson (2012) recently proposed that the occurrence of learning disabilities depends on such an interaction between specific and general cognitive factors. If a specific impairment, say in phonological or number processing, can be compensated by central executive resources, there is a smaller probability that the individual develops a learning disability. Otherwise, if executive processing resources are not sufficient to compensate or automatize basic cognitive processes, difficulties persist. This hypothesis has been explored in another report, investigating two cases of math learning difficulties (Haase et al., in press, this issue). In one case, math learning difficulties were associated with a lack of automatization and in the other case with impaired executive working memory resources.

There have been few studies that directly addressed the relationship between verbal memory and phonemic awareness during the performance of arithmetic tasks. Leather and Henry (1994) claim that both constructs share a certain amount of variance with arithmetic performance because phonemic manipulation demands arithmetical processes (for instance, phoneme elision tasks require, literally, the subtraction of a sound) and also involve working memory for the mental retention and management of verbal information. Phoneme elision tasks require both storage and processing of phoneme units because children usually hold the word in mind while deleting one sound and producing the new word with what is left (Oakhill and Kyle, 2000). Hecht et al. (2001) longitudinally investigated the role of phonological awareness in arithmetic development of children from different age ranges and found that from the 3rd to 4th grades, as well as from the 4th to 5th grades, this was the only subcomponent of phonological processing that explained the growth of performance in a standardized arithmetic task. According to the authors, the same memory resources engaged in arithmetic problem solving are also recruited in phonological awareness tasks.

Our findings are in accordance to what was reported by Michalczyk et al. (2013). The authors also found that the simultaneous inclusion of verbal and visuospatial working memory, the central executive as well as phonological awareness in a regression model showed that only phonological awareness—none of the working memory subcapacities—had a direct impact on basic quantity-number competencies. In this study, they investigated the performance of children aged 5 and 6 in a number sequence task, in which children had to recite the number word sequence forwards up to 31 and backwards from 5. Afterwards they had to name 3 subsequent and 3 preceding number words. Even though they did not use a transcoding task, one can infer from this result that phonological awareness might mediate the relation between verbal working memory and number words knowledge. Nevertheless, as mentioned above, our study was the first one to provide evidence regarding the mediation of the effect of verbal working memory on number transcoding by phonemic awareness.

### Final remarks

Mathematics encompasses a range of several different competences, such as numerical estimation, word problems, fact retrieval and number transcoding. Standardized arithmetic tasks usually assess these different abilities simultaneously and do not tap their specificities. It is important to investigate the distinct cognitive mechanisms that are associated with each of these mathematical skills. In our study, we concluded that phonemic awareness and verbal memory are directly connected to number transcoding, being important pathways between the verbal input and the transcription of the Arabic output.

The acuity of number sense, as measured by the Weber Fraction, did not influence number writing, suggesting that the assessment of numerical magnitude is not a necessary step in number transcoding. The acuity of number sense has been considered an important predictor of arithmetic performance (Halberda et al., 2008), but its relationship to number transcoding is less explored.

Although we did not explicitly assess children with learning disabilities, our results provide additional support to the hypothesis that phonemic awareness might be a cognitive mechanism that underlies both dyslexia and dyscalculia. Epidemiological studies describe high comorbidity rates between reading and mathematical difficulties: approximately 40% of dyslexics also have arithmetical difficulties (Lewis et al., 1994), and the prevalence of dyslexia and dyscalculia is similar, approximately 4–7% (Dirks et al., 2008; Landerl and Moll, 2010). The finding that phonemic awareness is related to number transcoding is useful in the comprehension of mathematical difficulties presented by dyslexic children (Haase et al., in press, this issue). We suggest that this should also be assessed in neuropsychological evaluations as well as in clinical interventions for children with learning disabilities.

### Conflict of interest statement

The authors declare that the research was conducted in the absence of any commercial or financial relationships that could be construed as a potential conflict of interest.
